# Physicians’ perceptions and preferences for implementing venous thromboembolism (VTE) clinical practice guidelines: a qualitative study using the Theoretical Domains Framework (TDF)

**DOI:** 10.1186/s13690-022-00820-7

**Published:** 2022-02-15

**Authors:** Juliana Abboud, Abir Abdel Rahman, Niaz Shaikh, Martin Dempster, Pauline Adair

**Affiliations:** 1 Centre for Improving Health Related Quality of Life, School of Psychology, Queens University Belfast, David Keir Building, 18-30 Malone Road, BT9 5BN Belfast, United Kingdom; 2 Rashid Hospital, Dubai Health Authority, Umm Hurair II 315, PO Box 4545, Dubai, United Arab Emirates; 3 Department of Medical Laboratory Sciences, Faculty of Health Sciences, University of Balamand, Youssef Sursok Street, PO Box 166378, Ashrafieh, Beirut, Lebanon

**Keywords:** Thromboprophylaxis, Venous thromboembolism, VTE risk assessment, Prophylaxis, Theoretical Domains Framework

## Abstract

**Background:**

Venous thromboembolism is a primary cause of morbidity and mortality in hospitalised patients. Clinical practice guidelines were developed to prevent venous thromboembolism events. This study adopted the Theoretical Domains Framework to explore the beliefs and perceptions of physicians adoption of clinical practice guidelines for the uptake of venous thromboembolism prevention guidelines.

**Methods:**

Semi-structured interviews were conducted with a stratified purposive sample of internal medicine physicians in an acute hospital. The interview topic guide was developed using the Theoretical Domains Framework to identify the factors perceived to influence the practice. Two researchers coded the interview transcripts using thematic content analysis. Emerging relevant themes were mapped to TDF domains.

**Results:**

A total of sixteen medical physicians were interviewed over a six-month period. Nine theoretical domains derived from thirty-three belief statements were identified as relevant to the target behaviour; knowledge (education about the importance of VTE guidelines); beliefs about capabilities (with practice VTE tool easier to implement); beliefs about consequences (positive consequences in reducing the development of VTE, length of stay, financial burden and support physician decision) and (negative consequence risk of bleeding); reinforcement (recognition and continuous reminders); goals (patient safety goal); environmental context and resources (workload and availability of medications were barriers, VTE coordinator and electronic medical record were enablers); social influences (senior physicians and patient/family influence the VTE practice); behavioural regulation (monitoring and mandatory hospital policy); and nature of the behaviour.

**Conclusions:**

Using the Theoretical Domains Framework, factors thought to influence the implementation of VTE clinical practice guidelines were identified which can be used to design theoretically based interventions by targeting specific psychological constructs and linking them to behaviour change techniques to change the clinical practice of physicians.

**Supplementary Information:**

The online version contains supplementary material available at 10.1186/s13690-022-00820-7.

## Background

Venous thromboembolism (VTE) is a primary cause of morbidity and mortality in hospitalised patients. Evidence-based clinical practice guidelines have been developed to prevent venous thromboembolism, outlining the recommendations for conducting VTE risk assessment and prescribing appropriate prophylaxis to prevent venous thromboembolism (VTE) in hospitalised patients [1–4].

A VTE prevention guideline consists of a VTE risk assessment, a risk of bleeding assessment, and clinical decision making on prophylactic choices based on the combination of VTE and bleeding risk factors. Several studies revealed that hospitalised patients at risk of VTE did not receive appropriate prophylaxis, and prophylaxis was prescribed less to medical patients than surgical patients [5–7]. Moreover, VTE risk assessment was not consistently undertaken for medical patients, and thus, appropriate prophylaxis was not always received by such patients [5–7].

A number of factors can influence the uptake of an evidence-based intervention. A systematic review explored barriers that affect physicians’ use of VTE guidelines, and the identified reasons were classified under three categories; costs and priorities, lack of role identification and practice culture [8]. A further systematic review, published around the same time by Khan and colleagues, revealed that a wide variety of interventions had been used to increase the rate of appropriate prophylaxis prescribed for patients at risk of VTE, such as alerts, education and multifaceted interventions. It was also reported that most of the interventions followed in these studies were effective at increasing the appropriate prophylaxis; however, the effectiveness level was different between studies [9]. Moreover, it was revealed in the updated systematic review, including RCT studies, that alert interventions involving a computer, and human alerts are more effective than multifaceted interventions in increasing the appropriate prophylaxis prescriptions and decreasing the incidence of VTE at risk hospitalised medical and surgical patients [10]. Furthermore, studies derived from behaviour change theory to inform the intervention to increase the uptake of VTE guidelines in medical patients were not identified in our systematic review study [11]. Thus, both our research and others have identified a need to explore the VTE guidelines regarding the uptake of this behaviour from a behaviour change theory-based perspective.

Changing the existing practice of physicians requires an understanding of the barriers and facilitators that affect their ability and decision to follow the clinical practice guidelines. Moreover, using a theoretical approach to identify these factors increases the likelihood that the interventions will be effective by targeting relevant mediators of change and identifying appropriate change strategies [12–14].

Many psychological theories have been adopted to explain health care providers’ behaviours. The Theoretical Domains Framework (TDF) is a framework that was designed and used in healthcare settings to investigate the influences on healthcare providers’ behaviour and inform interventions to change their behaviour around implementing evidence-based practices [15, 16]. It was initially developed by a multidisciplinary group of experts, including; psychological health theorists, health researchers and health psychologists. The first version of TDF contained 12 theoretical domains derived from 33 theories and 128 key theoretical constructs, and after the validation process, the refined TDF version included 14 domains and 84 theoretical constructs [16]. The TDF domains and constructs are outlined in (Additional file 1).

Many research studies have applied the TDF to identify the influences on healthcare provider behaviours to implement evidence-based guidelines, explore the barriers and facilitators towards this [17–19] and target interventions to implement the intended behaviours that will lead to better implementation [20]. Moreover, the TDF was used to design behaviour change interventions targeting clinicians [17, 20–22]. TDF has been extensively cited in the literature; thus, it is a well-established model for conducting research in this area [23].

This qualitative study used the Theoretical Domains Framework (TDF) to examine and identify the beliefs and perceptions as well as practices of physicians towards implementing VTE clinical practice guidelines in hospitalised medical patients.

The aim of this study was: (1) to explore beliefs that physicians hold about following the Venous thromboembolism clinical practice guidelines in hospitalised medical patients; (2) to identify the factors that influence and optimise physicians’ adherence to VTE guidelines when treating hospitalised medical patients. Moreover, the findings from this study will be potentially useful in the development of interventions to enhance the uptake of evidence into practice and improve the care of patients at risk of developing VTE.

Based on our knowledge, this is the first study to address factors that may influence Internal Medicine physicians’ behaviours in conducting VTE risk assessment and ordering recommended prophylaxis by using the TDF. It is a significant area to explore, as most of the research studies in this area have indicated that medical patients are less likely to receive the recommended VTE risk assessment and appropriate prophylaxis [5–7, 24]. In addition, a systematic review [11] highlighted the need to identify behaviour change interventions which target physicians clinical practice to increase the implementation of VTE guidelines.

## Methodology

### Design

This was a qualitative study using semi-structured face-to-face interviews and thematic content analysis of the interview transcripts, based on the Theoretical Domains Framework [23]. It was conducted with physicians treating medical hospitalised patients.

This study was reported based on the COREQ (Consolidated Criteria for Reporting Qualitative Research) checklist for comprehensive reporting of qualitative studies [25] (see additional file 2).

### Participants

Participants were recruited from physicians classified under internal medicine speciality in a 600+ beds acute hospital and providing medical care for hospitalised patients.

### Sampling Strategy

Participants were selected using stratified purposive sampling technique [26] to identify the different points of view and perceptions towards VTE guidelines implementation as well as to detect common beliefs. First, we purposefully sampled potential internal medicine physicians who were identified and selected because they were considered to represent a broad range of perceptions and attitudes towards VTE guidelines implementation practice. Then, we stratified the physicians by job title since we aimed to recruit physicians with different seniority levels, Consultant, Senior Specialist Registrar, Specialist Registrar and Resident to ensure all beliefs were captured. We aimed to recruit 16 participants to achieve appropriate thematic saturation [27]. Interviews would be stopped before this number if no new information was provided by participants, indicating data saturation [28]. Data saturation was discussed and agreed upon by (JA, AA and PA) before stopping the interviews.

### Recruitment

After the required ethics permission had been granted, potential participants were contacted first via telephone to explain the study purpose and procedure and check that participants met the research inclusion criteria. Once the eligible participants expressed their interest in participating, the information sheet and the consent form were sent via email. The information sheet outlined the purpose of the research, criteria for selection participants, the procedure adopted in this study, information on risk, and confidentiality. After giving their consent, the participants were invited for an interview. The interviews’ date, time, and location were based on participant preferences and carried out at the participants’ workplace, where no other person was present besides the interviewer. One researcher (JA), who received training on interview skills, conducted all the interviews over a six-month period from January to June 2019. The researcher (JA) introduced herself as a PhD student and conducted all interviews in English using the interview topic guide.

### Data collection

#### Interview Topic Guide

The clinical behaviours of interest were specified as “Behaviour 1) conducting VTE risk assessment for hospitalised medical patients and Behaviour 2) ordering recommended prophylaxis”. Since these two behaviours are those that affect the implementation of VTE guidelines, VTE risk assessment conducted following the admission of the patient and ordering prophylaxis is based on the VTE risk score and the risk of bleeding as per the VTE clinical practice guidelines [2–4]. An interview topic guide (see additional file 3) was developed based on the 14 theoretical domains of the TDF to elicit beliefs about each domain and identify the role of the domain in influencing the behaviour of physicians [23]. Each domain was linked to a group of questions that were used to explore the target behaviour “conducting VTE risk assessment and ordering the recommended prophylaxis”. The clinician (NS) within the research team reviewed the questions to ensure relevance to the topic under investigation.

The interview guide was pilot tested with two physicians to check the clarity and relevance of the questions, and modifications were made based on the feedback since it was relevant to the study question. Subsequent piloting was undertaken with another two physicians to ensure that the modifications were clear. Interviews were recorded, transcribed verbatim and anonymised, and field notes were made throughout and utilised during the initial coding identification. Interviews were not repeated, and transcripts were not returned to participants for comments, although they were available if requested.

### Analysis

Thematic analysis was undetaken using an inductive and deductive analytical approach to ensure all behavioural determinants were identified as well as detecting relevant influences that did not fit within the TDF domain [29].

Inductive analysis was initially conducted in line with a thematic analysis methodology [29]. One researcher (JA) transcribed the interviews and read them several times while taking notes to gain familiarity with the data and generate the belief statements. Independently another author (AA) analysed a sample of the transcripts (six) to confirm the generated beliefs and the codes which were generated from the belief statements. After that, the codes were categorised into overarching themes by the first author. The themes were discussed and agreed upon by three of the authors (JA, AA and PA). Subsequently, a deductive analysis was conducted independently by the two researchers (JA, AA) to assign the themes to the domains of the Theoretical Domain’s Framework [15, 16].

The two researchers (JA, AA) resolved disagreements by discussion of responses coded into the different domains. Discrepancies were addressed by a discussion with the third reviewer (PA). If consensus was not reached, the response was assigned to the domains identified by both researchers. Reliability between the two researchers was calculated by simple percentage agreement to measure consistency in coding within and across domains [30].

An initial coding guideline was developed following the analysis of the first two interviews. It was refined and updated during data collection as analysis progressed and new themes emerged.

The last step involved identifying the relevant theoretical domains for changing the behaviour of physicians.

### Identifying relevant theoretical domains

Theoretical construct domains were considered relevant if they met the following criteria similar to published studies: (1) relatively high frequency of specific beliefs and themes; (2) presence of conflicting beliefs; and (3) evidence of strong beliefs that may affect the target behaviour [20, 31].

Domains were identified as relevant after consensus discussion between the two researchers (JA, AA) and confirmed by the health psychologist within the team (PA).

Findings from the interviews are reported in tables as well as text to provide a clear description of the influences on the adherence to VTE clinical practice guidelines by conducting a VTE risk assessment for hospitalised medical patients and ordering the recommended prophylaxis. Quotations from transcripts, beliefs statements generated from these quotations, frequency counts for identified themes are presented in tables. Each belief statement was counted once within each interview to generate a frequency count across all interviews. Quotes were selected which best represented each of the themes, labelling each by physician category to protect anonymity.

### Research team and reflexivity

All authors are working within the academic and health care sector and to establish trustworthiness and strengthen the validity of the study findings, they followed the following: To ensure credibility in the data, the author (JA) paid attention when conducting the interviews to adopt a non-judgmental position whilst being aware that her position at the organisation could affect her interaction with the interviewees. Thus, the interview topic guide was followed closely during the interviews. Moreover, the interviews were conducted with different categories of physicians (i.e. Consultant, Senior Specialist Registrar, Specialist Registrar and Resident). Two researchers (JA, AA) used a coding manual to code and analyse the interview responses, and inter-rater reliability was calculated. One author (PA), who is experienced in conducting research studies, continuously monitored and reflected on the interview process and analysis to ensure the analysis was always a true reflection of the data.

To confirm transferability, the authors described the findings and supported the descriptions with quotes from the interviews. To enhance dependability and confirmability, the Theoretical Domains Framework was followed in conducting this study and a coding manual was developed [32, 33].

## Results

### Participants

Interviews were held over a six-month period. The interviews lasted between 20 and 62 min (M = 34 min, SD = 12 min). Sixteen participants were interviewed (5 male; 11 female), two consultants, five senior specialist registrars, seven specialist registrars and two residents were recruited to participate in the study. The physicians’ experience at the hospital ranged from 1 to 20 years and physicians aged 24-55 years. Thematic saturation was reached after interviewing 16 participants when the collected data did not add any new information to the study [28].

Forty beliefs from the 16 interviews were coded into the TDF domains. All belief statements supported by responses made in the interviews within each theoretical domain are reported in (additional files 4 and 5).

Interrater agreement for the coding between the two coders was calculated for four randomly selected interviews for all 14 domains. The overall agreement was 81%. It ranged between 50 and 100% at the domain level. An agreement was reached when the two coders identified the same response and allocated it to the same domain. Even though the interrater agreement was calculated, all disagreements between researchers were resolved through discussion and consensus during the coding process was agreed.

### Domains identified to be relevant

Nine theoretical domains relevant to the TDF were identified: knowledge, beliefs about capabilities, beliefs about consequences, reinforcement, goals, environmental context and resources, social influences and behavioural regulation, and nature of the behaviour.

A total of thirty-three belief statements were identified from the nine relevant domains of the TDF. The belief statements, corresponding TDF domains and representative quotes are summarised in (additional file 4). Quotes were selected from the responses of physicians from different seniority levels, Consultant (C), Senior Specialist Registrar (SSR), Specialist Registrar (SR) and Resident (R) to provide a representative perspective across the profession.

### Knowledge

Almost all participants were aware of VTE guidelines: “*Yes we are using hospital guidelines for risk assessing the patients and put them on the prophylaxis accordingly*” (P10 C); however, some participants thought that the VTE guidelines were not clear in certain clinical conditions to guide their practice: “*Sometimes I feel they are not very clear (guidelines). At some point, they are not matching the patient’s actual parameters*” (P3 S). Moreover, other participants stated that the availability of limited information about patient medical condition might affect completing the VTE risk assessment, mainly when patients were unconscious or without any escort: “*inadequate information, if the patient comes unconscious, we know nothing. It is difficult to start the patient on antibiotic prophylaxis without knowing the risk assessment*” (P8 SS). On the other hand, all participants mentioned that education and information about the importance of VTE guidelines, presenting real case scenarios and supported by data will improve the target behaviour. Thus, the *knowledge domain* was identified as potentially relevant.

### Beliefs about capabilities

The majority of participants were confident about performing the VTE risk assessment and ordering the recommended prophylaxis. All participants found that the VTE guidelines were easy to implement since the risk assessment tool has points, and based on the VTE risk score, the recommended prophylaxis will be ordered: “*Because we have these points, it is easy and clear”* (P7 S). Also, some elaborated that with practice, the VTE assessment tool became easier to implement: “*Now I know all points so within one minute I can finish it. With practice, it is easier*” (P7 S). This prompted us to select the *beliefs about capabilities* to be relevant domain.

### Beliefs about consequences

*Beliefs about consequences* were relevant since all participants identified a number of different benefits and risks that potentially influenced the target behaviour. Among the perceived benefits, almost all participants reported that following the VTE guidelines would reduce the development of DVT and PE and the morbidity and mortality cases (n=4) *“it will protect patients from developing DVT or PE, it will reduce the mortality & morbidity rate”* (P7 S). Moreover, it would decrease the financial burden on both the hospital and patient through; eliminating unnecessary medical tests: *“a waste of resources and then you have to do more advanced management for these patients”* (P8 SS); protecting the hospital reputation: *“it is a very good thing for our hospital reputation”* (P14 SS) and reducing hospitalisation days and management: *“shorten the hospital stay”* ( P10 C).

Furthermore, most participants highlighted that VTE guidelines supported and protected their clinical decision: *“They are guidelines to guide us”* (P2 SS), *“this guideline will protect me”* (P7 S). On the other hand, many participants reported that the target behaviour could be affected in complicated cases where there is a risk of bleeding associated with ordering prophylaxis: *“….in complicated cases in which the bleeding risk is high, it becomes difficult to decide should or should not prescribe prophylaxis*” (P6 SS).

### Reinforcement

When participants were asked about rewards needed to reinforce the VTE guidelines implementation, some participants stated that there was no need to give any rewards or incentives to target behaviour: “*Why rewards, it is part of our job*” (P7 S). Although, other participants thought that recognition, by highlighting the best performance: “*we can highlight the best performance*….” (P10 C) and continuous reminders and encouragement would reinforce the target behaviour: *“Continuous reminders during the rounds … encourages us”* (P9 S). The *reinforcement domain* was selected as relevant due to the evidence of a strong belief that may influence the behaviour.

### Goals

Almost all participants thought that performing the target behaviour would support the common healthcare goal of patient safety improvement: *“VTE prophylaxis is one of the patient safety parameters required by any institute”* (P1 C). This resulted in the selection of *goals* domain as relevant.

### Environmental context and resources

The *Environmental context and resources* domain was indicated as relevant since the majority of participants referred to various environmental factors that affected the target behaviour. Many participants identified the workload, including the number of patients they have to assess in a specific time, one factor that affected the target behaviour of conducting the VTE risk assessment: “*sometimes admitting doctors are very busy and they are not able to do the risk assessment” (P6 SS).*

In addition, few participants stated that the availability of mechanical prophylaxis affected their decision in ordering the appropriate prophylaxis, and some participants mentioned that in certain situations when a patient was admitted under a different speciality, the VTE risk assessment was missed: *“If it is my patient, I would. If the patients are not under me, I will not be doing the risk assessment. We can recommend”* (8 SS).

On the other hand, most participants indicated that having the VTE form as part of the electronic medical record facilitated the implementation of the VTE guidelines: “*I think it is quite convenient now because with the electronic system everything is there. You only have to check select or deselect*” (P3 S). Moreover, some participants thought that the availability of the VTE coordinator or nurse could facilitate the target behaviour: “*another professional or nurse could do the risk assessment, and we just need to verify it then it would be easier for us”* (P2 SS).

### Social influences

The *social influences* domain identifies whether other medical team members and patients’ relatives may influence physicians ordering the recommended prophylaxis. The majority of participants indicated that they discussed the VTE recommendations with their team members: “*we take multidisciplinary decisions to make better care*” (P13 S). In addition, participants indicated that the seniors from the clinical team had an impact on their behaviour and they might change their prophylaxis order based on the discussion with the senior: “*During the round, for example, while discussing with our consultants the type of the DVT prophylaxis might be changed*” (P7 S). Moreover, participants stated that they seek the opinion of an expert in the field.

Furthermore, some participants stated that their decision was affected by the patient and family level of awareness about the VTE risks and refusal of the prophylaxis treatment: “*Sometimes there are patients who refuse, that affects your decision for ordering prophylaxis*” (P16 R).

### Behavioural regulation

The *behavioural regulation* domain was identified to be relevant since participants identified various recommendations on how to regulate and influence physicians to perform the target behaviour. Monitoring the compliance to VTE guidelines and sharing the results: “*Leadership should monitor our compliance*” (P7 S), as well as linking VTE guidelines compliance to physicians’ performance evaluation: *“if the administration wants to be very strict about it, maybe they have to include in the Individual performance evaluation”* (P11 S) would induce the implementation of the VTE guidelines.

Moreover, as per many participants making VTE guidelines a mandatory policy: “*it is a part of the hospital policy which should be done*” (P8 SS) would support the target behaviour. However, two senior participants, a consultant level, had a contradicting point of view, they thought that too many regulations and restrictions might affect physicians’ role and autonomy: “*……When you say restrictive and make it mandatory physicians feel like you are taking away their autonomy*” (P10 C).

### Nature of the behaviour

Nature of the behaviour was derived from inductive analysis of the transcripts and added to the TDF as an extra domain. The interview responses revealed different opinions and conflicting viewpoints related to the VTE practice target behaviour. The majority of participants stated that they assessed all their patients for VTE risk: “*for all my patients I do VTE risk assessment”* (P2 SS). However, other participants revealed that they did not do a VTE risk assessment for all their patients: “*It is not 100% followed”* (P6 SS), few participants out of those who mentioned initially in the interview that they do VTE risk assessment for all, through the subsequent drill-down questions informed that they did not. On the other hand, some participants identified that they ordered prophylaxis without conducting VTE risk assessment: “*I am comfortable enough to start the DVT prophylaxis even without filling the scoring system”* (P14 SS). Moreover, other participants mentioned that they prescribed prophylaxis regardless of the VTE risk score since they followed their clinical judgement: “*If it is a young patient and unconscious, usually I am giving prophylaxis regardless of the score*” (P7 S), “*I follow my own judgment*” (P8 SS).

### Domains identified to be not relevant

Other theoretical domains appeared to be less relevant to the perceptions and preferences of physicians when making decisions about following the venous thromboembolism (VTE) clinical practice guidelines. They were skills, social/professional role and identity, optimism, intentions, memory attention and decision processes and emotion. The belief statements, corresponding TDF domains and representative quotes are presented in (additional file 5).

### Skills

The *Skills* domain was not found to be challenging as physicians repeatedly reported that the behaviour related to the following VTE guidelines did not require any particular skill rather clinical knowledge on conducting the general medical assessment. Most of the participants believed that as long as they had a basic medical background and were adequately trained to take a patient history and conduct the clinical assessment, then they had enough skills to conduct VTE risk assessment and make the appropriate prophylaxis recommendations: “*It is part of patient’s general assessment* (P5 SS). *It takes good history skills, good physical examination skills, and it should include a good clinical judgment and be able to decide*” (P9 S).

### Social/professional role and identity

*Social/professional role and identity* was identified as an irrelevant domain since most of the participants identified the target behaviour as part of their professional role and job: “*It is part of our job*” (P7 S).

### Optimism and Intentions

*Optimism and Intentions* Domains were identified as not relevant for performing the target behaviour because responses in these domains revealed a low frequency of beliefs statements.

#### Memory, attention and decision processes

The majority of the participants reported that forgetting to perform the target behaviour was not a concern for them since using a tool related to VTE guidelines practices facilitates attention to detail steps to follow: “*We have the VTE assessment form*” (P6 SS). Moreover, physicians were familiar with the tool itself, and no particular attention or specific decision processes were needed since they just had to follow the form and tick the required boxes: “*it is just a series of questions tick boxes that need to be done, and then you provide the necessary prophylaxis*” (P10 C). In addition, participants stated that the VTE form was an online chart within the patient admission process: “*We usually have an online chart for VTE risk in the admission package”* (P15 R).

#### Emotion

On the other hand, most interviewed participants stated that their own emotions would not influence whether they followed the VTE guidelines or not. However, some participants revealed that they were happy and satisfied to implement the VTE guidelines since they prevented causing harm to the patients: “*For me as a physician, I feel happy and safe that I am preventing the patient from getting any life-threatening condition or morbidity or mortality in the hospital*” (P12 S).

#### Important factors identified that do not fit within the TDF domain

Themes that did not fit within the TDF domains were also reported in this study and, therefore, important to include for comprehensiveness. Some participants reported that they sometimes ignored the electronic alerts that they received to complete the VTE risk assessment tool if missed. “*To be very honest that it happens that we overlook the warning that is coming to us also. Although, we know that we have to do it” (P6 SS)*. Moreover, the Language barrier was highlighted as one of the factors that affect patient care management when the patient and the physician do not speak the same language. Furthermore, the electronic medical record use by physicians with older age groups was highlighted as a limitation since they spent extra time to complete the required documentation electronically. “*I think the younger ones are faster at typing. I am not as fast as their reaction time, it might be a limitation*” (P10 C).

## Discussion

The VTE clinical practice was explained, in this study, by a set of determinants (barriers & facilitators) (additional file 6) informed by the Theoretical Domains Framework perceived to be influencing physicians’ behaviour towards VTE guidelines implementation, through conducting VTE risk assessment and ordering appropriate prophylaxis among internal medicine physicians. Nine domains were identified as being relevant to VTE guidelines practices, including knowledge, beliefs about capabilities, beliefs about consequences, reinforcement, goals, environmental context and resources, social influences and behavioural regulation, and nature of the behaviour.

While each physician’s experience was unique, participants reported similar patterns and approaches to support the uptake of VTE guidelines. They concurred with the importance of these determinants to alter the behaviour of physicians, such as (1) providing information about the importance of VTE guidelines and health consequences; (2) implementing a VTE mandatory policy; (3) introducing a user-friendly automated VTE risk assessment tool as part of the admission process including scores linked to automatic order sets for prophylaxis; (4) social support, teamwork and encouragement by senior physicians and being the role model for junior physicians; (5) having a VTE coordinator; (6) setting outcome goals and including them in the performance evaluation; (7) monitoring and providing evaluative feedback on performance to physicians; (8) introducing reminders in the morning rounds and discussions during staff meetings.

Education interventions proved to improve the VTE target behaviour, mainly when accompanied by multicomponent interventions [9, 10]. However, the effectiveness level varied between the educational categories while enforcing the clinical practice guidelines [34, 35]. Social influence interventions, including interaction in small-group meetings and multi-professional collaboration and teamwork, mainly were effective towards guidelines implementation [34, 35]. Having a dedicated coordinator for VTE guidelines facilitated the target behaviour as identified in other research studies [36, 37].

Consistent with the published literature, the qualitative interviews highlighted that decision support strategy, including alerts, reminders, and computerised decision support, would facilitate the implementation of VTE prevention guidelines [8, 38, 39]. Nevertheless, the interviews noted that physicians might ignore alerts consistent with other published literature [40], which might contribute to alert fatigue [41]. Considering their potential effect on altering physicians’ behaviour and associating with higher proportions of patients who received prophylaxis highlights a need to explore this area in future research studies [10].

Although physicians’ beliefs were predominately positive in favour of the target behaviour, they had reported some barriers such as (1) unclear clinical practice guidelines recommendations for prophylaxis management in certain clinical conditions; (2) the risk of bleeding due to prophylaxis treatment; (3) workload pressure and competing tasks; and (4) undefined responsibilities about completing the risk assessment.

Similar to what was highlighted in other studies, lack of awareness about the necessary treatment followed when a patient has contraindications was identified as one of the barriers [8]. Physicians were reluctant to prescribe prophylaxis due to the probability of developing complications similar to what was highlighted in other studies. Patients’ factors related to the risk of bleeding were identified as barriers that prohibited physicians from following the guidelines due to the belief that it might cause adverse events [8, 42]. Repeatedly, the workload was highlighted as one of the barriers that prevented physicians from following the clinical practice guidelines [43].

Another key factor that affected the target behaviour was related to patient and family attitude towards VTE prophylaxis treatment. Patient and family involvement in care management is essential to improve care delivery [44]. Moreover, based on the literature, patients need to know about the VTE symptoms, risk factors, and complications associated with harm [45]. In addition, informing patients of the risks and benefits of prophylaxis may decrease the refusal rate of VTE treatment but would require a deeper understanding of this phenomenon [46].

In the design and implementation of future interventions to change the VTE practice target behaviour, there is a need to link the identified behaviour change determinants to interventional components behaviour change techniques (BCTs) to specific mechanisms of action affecting behaviour change [14, 47]. The selection of specific BCT differs by type of behaviour [48]. The most frequent BCTs in the implementation interventions were feedback on behaviour, instruction on (how) to perform the behaviour, social comparison, credible source, prompts/cues, and goal setting (behaviour). In the de-implementation interventions, the most frequent BCTs were feedback on behaviour, instruction on (how) to perform the behaviour, social comparison, feedback on behaviour outcomes, and information about social and environmental consequences [49].

### Strengths and limitations

This study is the first to explore internal medicine physicians’ beliefs and perceptions about implementing VTE prevention guidelines to the best of our knowledge. Coding and analysing the interviews responses by two researchers (JA, AA) using a coding manual helped achieve a high agreement. It decreased the individual interpretations of the content of interview responses. Moreover, using the Theoretical Domains Framework was a strength since the study utilised theory to identify the relevant factors that influence the target behaviour. This guided the identification of evidence-informed interventions to increase the uptake of VTE guidelines practices. Furthermore, this study followed both inductive and deductive analysis and presented both TDF and non-TDF-related findings to overcome the overlook of factors identified during the interviews that do not fit within TDF domains [50].

While this study has provided significant insight into the factors that may influence VTE practice, The findings should be considered in the context of the study’s limitations to one clinical speciality, internal medicine. The identified perceived influences are the opinions of the interviewed physicians about their practice and may not be the actual influences for other hospitals.

## Conclusions

The use of the TDF in this study delivers a theory-driven approach to identifying factors that are likely to influence physicians’ behaviour in VTE prevention guidelines practice.

The identified domains and themes can be utilised to develop a questionnaire to explore further the VTE guidelines in future quantitative studies. Moreover, the results can be used to design theoretically-based interventions by targeting specific psychological constructs and implementing behaviour change techniques to change the behaviour of physicians to increase the uptake of evidence into practice.

## Supplementary Information

**Additional file 1.** The TDF domains and constructs.

**Additional file 2.** COREQ checklist.

**Additional file 3.** Interview topic guide.

**Additional file 4.** Summary of belief statements and sample quotes assigned to the theoretical domains identified as relevant.

**Additional file 5.** Summary of belief statements and sample quotes assigned to the theoretical domains identified as not relevant.

**Additional file 6.** Barriers and facilitators.

## Data Availability

The dataset (which includes individual transcripts) is not publicly available due to confidentiality.

## Abbreviations

BCT: Behaviour change technique
COREQ: Consolidated Criteria for Reporting Qualitative Research
C: Consultant
DVT: Deep Vein Thrombosis
MRP: Most Responsible Physician
PE: Pulmonary Embolism
SR: Specialist Registrar
SSR: Senior Specialist Registrar
R: Resident
TDF: Theoretical Domains Framework
VTE: Venous Thromboembolism

## Declarations

All participants provided written informed consent before participating in the study, which included consent to publish anonymous quotes from individual participants.
