# Supplementary material for: Physicians’ perceptions and preferences for implementing venous thromboembolism (VTE) clinical practice guidelines: a qualitative study using the Theoretical Domains Framework (TDF)
Source: Arch Public Health. 2022 Feb 15;80:52. doi: 10.1186/s13690-022-00820-7 (PMC8845331; doi:10.1186/s13690-022-00820-7)
Supplement: Supplementary file 1 — Additional file 1. The TDF domains and constructs. [file 13690_2022_820_MOESM1_ESM.docx]

The Theoretical Domain Framework (adapted from Cane al 2012)

| **Domain Definition** | **Construct** |
| --- | --- |
| 1. **Knowledge**   (An awareness of the existence of something) | Knowledge (including knowledge  of condition/scientific rationale)  Procedural knowledge  Knowledge of task environment |
| 1. **Skills**   **(**An ability or proficiency acquired through practice) | Skills  Skills development  Competence  Ability  Interpersonal skills  Practice  Skill assessment |
| 1. **Social/professional role and identity**   (A coherent set of behaviours and displayed personal qualities of an individual in a social or work setting) | Professional identity  Professional role  Social identity  Identity  Professional boundaries  Professional confidence  Group identity  Leadership  Organisational commitment |
| 1. **Beliefs about capabilities**   (Acceptance of the truth, reality or validity about an ability, talent  or facility that a person can put to constructive use) | Self-confidence  Perceived competence  Self-efficacy  Perceived behavioural control  Beliefs  Self-esteem  Empowerment  Professional confidence |
| 1. **Optimism**   (The confidence that things will happen for the best or that  desired goals will be attained) | Optimism  Pessimism  Unrealistic optimism  Identity |
| 1. **Beliefs about Consequences**   (Acceptance of the truth, reality, or validity about outcomes of a behaviour in a given situation) | Beliefs  Outcome expectancies  Characteristics of outcome  expectancies  Anticipated regret  Consequents |
| 1. **Reinforcement**   (Increasing the probability of a response by arranging a  dependent relationship, or contingency, between the  response and a given stimulus) | Rewards (proximal/distal, valued/not  valued, probable/improbable)  Incentives  Punishment  Consequents  Reinforcement  Contingencies  Sanctions |
| 1. **Intentions**   (A conscious decision to perform a behaviour or a resolve to act in a certain way) | Stability of intentions  Stages of change model  Transtheoretical model and  stages of change |
| 1. **Goals**   (Mental representations of outcomes or end states that an  individual wants to achieve) | Goals (distal/proximal)  Goal priority  Goal/target setting  Goals (autonomous/controlled)  Action planning  Implementation intention |
| 1. **Memory, attention and decision processes**   (The ability to retain information, focus selectively on aspects of the environment and choose between two or more alternatives) | Memory  Attention  Attention control  Decision making  Cognitive overload/tiredness |
| 1. **Environmental context and resources**   (Any circumstance of a person’s situation or environment that discourages or encourages the development of skills and abilities, independence, social competence and adaptive  behaviour) | Environmental stressors  Resources/material resources  Organisational culture/climate  Salient events/critical incidents  Person × environment interaction  Barriers and facilitators |
| 1. **Social influences**   (Those interpersonal processes that can cause individuals to  change their thoughts, feelings, or behaviours) | Social pressure  Social norms  Group conformity  Social comparisons  Group norms  Social support  Power  Intergroup conflict  Alienation  Group identity  Modelling |
| 1. **Emotion**   (A complex reaction pattern, involving experiential, behavioural, and physiological elements, by which the individual attempts to deal with  a personally significant matter or event) | Fear  Anxiety  Affect  Stress  Depression  Positive/negative affect  Burn-out |
| 1. **Behavioural regulation**   (Anything aimed at managing or changing objectively observed or  measured actions) | Self-monitoring  Breaking habit  Action planning |
