# Supplementary material for: Physicians’ perceptions and preferences for implementing venous thromboembolism (VTE) clinical practice guidelines: a qualitative study using the Theoretical Domains Framework (TDF)
Source: Arch Public Health. 2022 Feb 15;80:52. doi: 10.1186/s13690-022-00820-7 (PMC8845331; doi:10.1186/s13690-022-00820-7)
Supplement: Supplementary file 2 — Additional file 2. COREQ checklist. [file 13690_2022_820_MOESM2_ESM.docx]

Interview topic Guide

**A qualitative study of physicians’ perceptions and preferences**

**when making decisions about following**

**the Venous thromboembolism clinical practice guidelines**

**Section A: Demographic Characteristics**

**1. What is your age?**

|  |  |
| --- | --- |
|  |  |
|  |  |

**2. Gender**

| Male | **🞏** |
| --- | --- |
| Female | **🞏** |

**3. How many Years of experience do you have at the current hospital?**

| **Less than 1 year** | **🞏** |
| --- | --- |
|  | **🞏** |
|  | **🞏** |
|  | **🞏** |

**4. What is your current Professional title or position?**

| **Consultant** | **🞏** |
| --- | --- |
| **Senior Specialist Registrar** | **🞏** |
| **Specialist Registrar** | **🞏** |
| **Resident** | **🞏** |

**Section B: The next set of questions are related to perceptions and beliefs**

| **Construct Domain** | **Interview Questions** | |
| --- | --- | --- |
| 1. **Knowledge**   (An awareness of the existence of something) | *I would like to find out about your knowledge and use of guidelines:*   - Do you use any clinical practice guidelines to assess medical patients for VTE risk and order recommended prohylaxis)? - How do you use the clinical practice guidelines? (i.e. what do you actually, physically do? - Can you describe what do you do, who is involved? Do you have any example in mind? - Are you aware about the content of the guidelines? tell me - Do you ever read the clinical practice guidelines to check if a behaviour you performed was guideline-compliant?) - What other evidence are you aware of, or do you use? Where do you find these information ? | |
| 1. **Skills**   **(**An ability or proficiency acquired through practice) | - *How easy or difficult would it be* to ***conduct VTE risk assessment for hospitalized medical patients and order recommended Prophylaxis***? Do you have ever find it difficult? - *What skills are required* to ***conduct VTE risk assessment for hospitalized medical patients and order recommended Prophylaxis***? - *Did you receive any specific training?* - *How often do you* ***conduct VTE risk assessment for hospitalized medical patients and order recommended Prophylaxis if required***? How many patients do you see per…? how many times do you conduct …. | |
| 1. **Social/professional role and**   **identity**  (A coherent set of behaviours  and displayed personal qualities  of an individual in a social or  work setting) | - *Do you sometimes feel constrained by clinical practice guidelines? Give me an example of a situation where you felt constrained?* - How does this affect your professional autonomy and your role as a physician to make choices and treatment decisions for your patients? - Is there anything else about your professional role that influences ***you conducting VTE risk assessment for hospitalized medical patients and ordering recommended Prophylaxis***? (i.e. consensus in your profession/ among colleagues, others opinions peers/superiors) | |
| 1. **Beliefs about capabilities**   (Acceptance of the truth, reality  or validity about an ability, talent  or facility that a person can put  to constructive use) | - What problems/difficulties do you think you might encounter in ***conducting VTE risk assessment for hospitalized medical patients and ordering recommended Prophylaxis***? Give me an example - How able do you feel to conduct the VTE assessment and order prophylaxis? - What would help you overcome these problems/difficulties? | |
| 1. **Optimism**   (The confidence that things will  happen for the best or that  desired goals will be attained) | - *How confident are you about doing this*? (that you can ***conduct VTE risk assessment for hospitalized medical patients and order recommended Prophylaxis,*** despite any difficulties) - *What makes you confident/ not confident?* | |
| 1. **Beliefs about Consequences**   (Acceptance of the truth, reality,  or validity about outcomes of a  behaviour in a given situation) | *What are the benefits* of ***conducting VTE risk assessment for hospitalized medical patients and ordering recommended Prophylaxis***?   - For you as a physician, for hospital? - what harms might be avoided to self, patients colleagues? long-term & short-term, financial.   *What are the disadvantages* of ***conducting VTE risk assessment for hospitalized medical patients and ordering recommended Prophylaxis***?   - what harms might there be to self, to patients?, colleagues, long-term & short-term, financial.   How does it matter to you to ***conduct VTE risk assessment for hospitalized medical patients and order recommended Prophylaxis***? | |
| 1. **Reinforcement**   (Increasing the probability of a  response by arranging a  dependent relationship, or  contingency, between the  response and a given stimulus) | *Are there any incentives* to encourage ***conducting VTE risk assessment for hospitalized medical patients and ordering recommended Prophylaxis***?  *Any rewards needed?* | |
| 1. **Intentions**   (A conscious decision to perform  a behaviour or a resolve to act in  a certain way) | How much to do you intend to ***conducting VTE risk assessment for hospitalized medical patients and ordering recommended Prophylaxis***?  What influences how much you intend to ***conducting VTE risk assessment for hospitalized medical patients and ordering recommended Prophylaxis***?  What drives you to comply with guidelines implementation? | |
| 1. **Goals**   (Mental representations of  outcomes or end states that an  individual wants to achieve) | - *How important do you feel it is* to ***conduct VTE risk assessment for hospitalized medical patients and order recommended Prophylaxis***? (in relation to other behaviours required to treat the patient). - *Tell me why?* | |
| 1. **Memory, attention and decision processes**   (The ability to retain information,  focus selectively on aspects of  the environment and choose  between two or more  alternatives) | *What thought processes might guide your decision* to ***conduct VTE risk assessment for hospitalized medical patients and order recommended Prophylaxis***? (what goes through your mind?)  How do you remember what to do ?   - In what situations, if any, might it be difficult to think of alternatives to conduct risk assessment? - Is ***conducting VTE risk assessment for hospitalized medical patients and ordering recommended Prophylaxis*** something you would usually do? |  |
| 1. **Environmental context and resources**   (Any circumstance of a person’s  situation or environment that  discourages or encourages the  development of skills and  abilities, independence, social  competence and adaptive  behaviour) | In what way is ***conducting VTE risk assessment for hospitalized medical patients and ordering recommended Prophylaxis*** *affected by different clinical situations*? (any other situations?)  What aspects of your clinical environment( physical vs resource factor) influence whether or not you ***conduct VTE risk assessment for hospitalized medical patients and order recommended Prophylaxis?***   - Are there any competing tasks or time constraints that influence whether you might ***conduct VTE risk assessment for hospitalized medical patients and order recommended Prophylaxis***? |  |
| 1. **Social influences**   (Those interpersonal processes  that can cause individuals to  change their thoughts, feelings,  or behaviours) | *Would other team members have a view* of you or an influence ***conducting VTE risk assessment for hospitalized medical patients and ordering recommended Prophylaxis***?   - who else? Other clinicians, medical staff, relatives? - What do you think those views might be? - How might the views of other team members affect you ***conducting VTE risk assessment for hospitalized medical patients and ordering recommended Prophylaxis***? How do you feel? - Do your colleagues generally agree with you ? |  |
| 1. **Emotion**   (A complex reaction pattern,  involving experiential,  behavioural, and physiological  elements, by which the  individual attempts to deal with  a personally significant matter  or event) | *Would you feel worried* about ***conducting VTE risk assessment for hospitalized medical patients and ordering recommended Prophylaxis***?   - If so, in what ways & in what kind of circumstances? - Describe your emotions / feelings as a physician - To what extent do you feel nervous? - How would it influence your work stress to ***conduct VTE risk assessment for hospitalized medical patients and order recommended Prophylaxis***?   Do patient emotions ever influence your decision? |  |
| 1. **Behavioural regulation**   (Anything aimed at managing or  changing objectively observed or  measured actions) | *If you’re thinking about changing your own practice, how would you do this*?   - What might you do in order to improve compliance with clinical practice guidelines - Are there procedures or ways of working that might encourage you to ***conduct VTE risk assessment for hospitalized medical patients and order recommended Prophylaxis***? - What might need to be done differently? - What would you do differently? - Who needs to do what differently when, where, how, how often and with whom? |  |
| 1. **Nature of the behaviour** | “The evidence from research suggests that conducting VTE assessment and prescribing Prophylaxis is variable. However, there is evidence to support a restrictive practice. With that in mind, in terms of **increasing the practice of VTE risk assessment**:”  • What might need to be done differently?  • What would you do differently?  • Who needs to do what differently when, where, how, how often and with whom?   - Would you be comfortable proceeding without conducting VTE assessment and prescribing Prophylaxis as per the guidelines recommendations? |  |
