# Supplementary material for: Physicians’ perceptions and preferences for implementing venous thromboembolism (VTE) clinical practice guidelines: a qualitative study using the Theoretical Domains Framework (TDF)
Source: Arch Public Health. 2022 Feb 15;80:52. doi: 10.1186/s13690-022-00820-7 (PMC8845331; doi:10.1186/s13690-022-00820-7)
Supplement: Supplementary file 3 — Additional file 3. Interview topic guide. [file 13690_2022_820_MOESM3_ESM.docx]

**Table of consolidated criteria for reporting qualitative studies (COREQ) checklist.**

| **Topic** | **Item No** | **Guide questions/description** | **Response** | **Reported on Page No.** |  |
| --- | --- | --- | --- | --- | --- |
| **Domain 1 : Research team and reflexivity** | | | |  |  |
| ***Personal characteristics*** | | | |  |  |
| Interviewer | 1 | Which author/s conducted the interview? | The primary author (JA) conducted the interviews. | 7 |  |
| Credentials | 2 | What were the researcher’s credentials? E.g. PhD | (JA) is a PhD student. |  |  |
| Occupation | 3 | What was their occupation at the time of the study? | Quality consultant | NA |  |
| Gender | 4 | Was the researcher male or female? | Female | NA |  |
| Experience & training | 5 | What experience or training did the researcher have? | (JA) received training at Queen’s University Belfast on Qualitative studies and conducting research interviews by | 7 |  |
| ***Relationship with participants*** | | | |  |  |
| Relationship established prior to  study commencement | 6 | Was a relationship established prior to study commencement? | Participants were unknown to the researcher before the  research start, they were contacted through telephone | 7 |  |
| Participant knowledge of the interviewer | 7 | What did the participants know about the researcher? | Participants were informed about the study prior to the interviews through a phone call by the interviewer and the information sheet that they had received. participants was informed that the study is part of interviewer’s PhD requirements | 7 |  |
| Interviewer characteristics | 8 | What characteristics were reported about the interviewer? e.g. Bias, assumptions, reasons and  interests in the research topic | The interviewer introduced herself as a PhD student  Research team and reflexivity was reported | 7 |  |
| **Domain 2: Study design** | | | |  |  |
| ***Theoretical framework*** | | | |  |  |
| Methodological orientation & theory |  | What methodological orientation was stated to underpin the study? e.g. grounded theory, discourse analysis, ethnography, phenomenology, content analysis | Thematic content analysis | 8 |  |
| ***Participant selection*** |  |  |  |  |  |
| Sampling |  | How were participants selected? e.g. purposive, convenience, consecutive, snowball | Stratified Purposive sampling | 6 |  |
| Method of approach |  | How were participants approached? e.g. face-to-face, telephone, mail, email | JA contacted potential participants by telephone and a brief summary of the study was given. Interviews were conducted face-to-face | 7 |  |
| Sample size |  | How many participants were in the study? | 16 | 11 |  |
| Non-participation |  | How many people refused to participate or dropped out? Reasons? | Not applicable: participation was voluntary. |  |  |
| Setting of data collection |  | Where was the data collected? e.g. home, clinic, workplace | In an office in the participants workplace | 7 |  |
| Presence of non-participants |  | Was anyone else present besides the participants and researchers? | Only the interviewer and the participant were present at  the interview | 7 |  |
| Description of sample |  | What are the important characteristics of the sample? e.g. demographic data, date | Sixteen participants were interviewed (5 male; 11 female), two consultants, five senior specialist registrars, seven specialist registrars and two residents. The physicians’ experience at the hospital ranged from 1- 20 years and physicians aged 24-55 years | 11 |  |
| ***Data collection*** |  |  |  |  |  |
| Interview guide |  | Were questions, prompts, guides provided by the authors? Was it pilot tested? | The interview topic guide was informed by TDF. The first two interviews were reviewed for interview technique. The topic guide was reviewed after each interview | 8 |  |
| Repeat interviews |  | Were repeat interviews carried out? If yes, how many? | No repeat interviews | 8 |  |
| Audio/visual recording |  | Did the researcher use audio or visual recording to collect the data? | All interviews were digitally recorded and transcribed by the interviewer | 8 |  |
| Field notes |  | Were field notes made during and/or after the interview? | Field notes were made throughout the interviews | 8 |  |
| Duration |  | What was the duration of the interviews? | The mean interview length was 34 min (Range 20-62 min). | 11 |  |
| Data saturation |  | Was data saturation discussed? | Data saturation was reached at interview number 16. | 11 |  |
| Transcripts returned |  | Were transcripts returned to participants for comment? | Transcripts were not returned to the participants, but they were available on request. | 8 |  |
| **Domain 3: analysis and findings** | | | | |  |
| ***Data analysis*** | | | | |  |
| Number of data coders |  | How many data coders coded the data? | Two coders coded the data. | 9 |  |
| Description of coding tree |  | Did authors provide a description of the coding tree? | Coding guideline was developed based on the theoretical domains. Authors met regularly to discuss interviews, coding and themes | 8 |  |
| Derivation of themes |  | Were themes identified in advance or derived from the data? | Themes were derived from the data by open coding and then mapped to the TDF. | 8&9 |  |
| Software |  | What software, if applicable, was used to manage the data? | No software was used. The analysis was done manually |  |  |
| Participant checking |  | Did participants provide feedback on the findings? | Participants were not invited to check the analysis | 8 |  |
| ***Reporting*** | | | | | |
| Quotations presented |  | Were participant quotations presented to illustrate the themes/findings? | Yes. Supporting quotations from participants were presented in the additional files 4 | additional files 4 &5 |  |
| Data and findings consistent |  | Was there consistency between the data presented and the findings? | Results of participants beliefs were embedded in text and quotes were used to illustrate the findings in participants own language | Results section  & additional files 4 &5 |  |
| Clarity of major themes |  | Were major themes clearly presented in the findings? | Major themes were presented in the results section and illustrated with quotes | Results section  & additional files 4 |  |
| Clarity of minor themes |  | Is there a description of diverse cases or discussion of minor themes? | Variations in views and themes and minor themes were  presented. | Results section and discussion section |  |

*TDF= Theoretical Domains Framework
