# Supplementary material for: Physicians’ perceptions and preferences for implementing venous thromboembolism (VTE) clinical practice guidelines: a qualitative study using the Theoretical Domains Framework (TDF)
Source: Arch Public Health. 2022 Feb 15;80:52. doi: 10.1186/s13690-022-00820-7 (PMC8845331; doi:10.1186/s13690-022-00820-7)
Supplement: Supplementary file 4 — Additional file 4. Summary of belief statements and sample quotes assigned to the theoretical domains identified as relevant. [file 13690_2022_820_MOESM4_ESM.docx]

| Summary of belief statements and sample quotes from physicians assigned to the theoretical domains identified as relevant | | | |
| --- | --- | --- | --- |
| Key Domain | **Themes** | Sample Quote | Frequency |
| **Knowledge** | I am aware of VTE guidelines | “Yes we are using hospital guidelines for risk assessing the patients and put them on the prophylaxis accordingly” (P10)  “The clinical practice guideline is in two parts. First part is to assess the risk of VTE and the second part is about the appropriate VTE prophylaxis for the patient according to the score that is calculated in the first part” (P1 C) | 15 |
|  | The recommended prophylaxis based on the identified risk score does not cover all patients’ clinical conditions | “Sometimes I feel they are not very clear (guidelines). At some point, they are not matching the patient’s actual parameters”(P3 S)  “Sometimes, clear recommendation is not there in those types of gray areas”( P5 SS)  “Sometimes the guideline is still vague with regards to decision. There is no clear guidelines for some patients we have”(P13 S) | 9 |
|  | Limited information about the patient affects completing the VTE risk assessment | “has no one with him to give us the history or the escort does not know the detailed history” (P7 S)  “inadequate information, If the patient comes unconscious, we know nothing. It is difficult to start the patient on antibiotic prophylaxis without knowing the risk assessment” (P8 SS)  “Sometimes in patients who are unable to communicate and have no relative present to give history, is difficult to score them” (P15 R) | 9 |
|  | Education and information about the importance of VTE guidelines will enforce implementing the VTE guidelines | “Education ,you have to mention real time cases who had excellent VTE risk assessment and orders compared to that had no real assessment or genuine assessment for risk factors and they had VTE in hospital or after discharge” ( P1 C)  “let them know more that how important it is because once the young doctor will know that it is important then they will go for it” (P5 SS)  “Talk with them, educate them, tell them about the risk and benefits” ( P12 S) | 16 |
| **Beliefs about capabilities** | It is easy for me to implement the VTE guidelines | “Because we have these points, it will be easier for everybody, it is easy, clear” ( P7 S)  “it is a user-friendly form and it is very easy to use” (P14 SS) | 16 |
|  | With practice the VTE risk assessment tool will be easier to implement | “I used to do it next day after admission, but now I am used to the system. Now I know all points so within one minute I can finish it. With practice, it is more easy” ( P7 S)  “I have been doing it for so long. It is the experience” (P10 C) | 9 |
|  | I am confident to perform the VTE risk assessment | “I am very confident, and I do it every day” ( P2 SS)  “Quite pretty confident” ( P10 C)  “Very confident” ( P11 S) | 13 |
| Beliefs about Consequences | Following VTE guidelines would reduce the development of DVT and PE | “it will protect patients from developing DVT or PE, it will reduce the mortality & morbidity rate” (P7 S) | 14 |
|  | Following VTE guidelines would decrease patient length of stay | “reduce the hospitalization days and the management” ( P7 S)  “ensure unnecessary prolonged patient stay in the hospital”  ( P8 SS)  “shorten the hospital stay” ( P10 C) | 7 |
|  | Following VTE guidelines would avoid financial burden for patient and hospital | “a waste of resources and then you have to do more advanced management for these patients” ( P8 SS)  “it will reduce the number of investigations” ( P7 S)  “From the insurance point of view it can become a medico-legal case” ( P5 SS)  “it is a very good thing for our hospital reputation” ( P14 SS) | 8 |
|  | Following VTE guidelines would support and protect the physician clinical decision | “They are guidelines to guide us” ( P2 SS)  “this guideline will protect me” ( P7 S)  “Guidelines will support your clinical practice” ( P6 SS)  “Clinical guidelines are very helpful and they guide us also to what is the best advice for the patient” (P1 C) | 14 |
|  | I am less confident to order the recommended prophylaxis in complicated cases where there is a risk of bleeding | “putting them on prophylaxis is a bit challenging for us, there are some complicated cases in which the bleeding risk is high and then it becomes difficult to decide should we or should not” ( P6 SS)  “Complications is one of the important concerns especially in high risk patients to develop complications like bleeding” (P4 S)  “So I feel difficulty, it comes when you do not have much information or if there is a contraindication” ( P12 S) | 13 |
| **Reinforcement** | No rewards are needed to implement the VTE guidelines | “I do not think there should be any specific rewards or incentives” ( P 10 C)  “Why rewards , it is part of our job” ( P7 S)  “Do we need any more incentives in treating a patient?”(P9 S) | 9 |
|  | Recognition from work context will support the implementation of the VTE guidelines | “we can highlight the best performance. this is more encouraging to hear rather than just being told that department X is not in compliance with VTE prophylaxis” ( P10 C) | 3 |
|  | Continuous reminders and encouragement will enforce the implementation of the VTE guidelines | “Continuous reminders during the rounds, during the discussions in the morning regarding the VTE risk assessment of the patient. Those things encourages us” ( P9 S)  “encourage for VTE prophylaxis everywhere so I am not restricting, but I am pushing for it” ( P1 C) | 12 |
| **Goals** | Patient Safety is a goal in health care | “It is important for patient safety” ( P6 SS)  “VTE prophylaxis is one of the patient safety parameters  required by any institute” ( P1 C)  “For the patient’s safety” (P16 R) | 14 |
| **Environmental context and resources** | I might not conduct the VTE risk assessment in case of high workload | “as a Medical on call and I am receiving one or two patients per hour so I did not have time to make this assessment properly” ( P7 S)  “the first medical on call overwhelmed with number of cases, it might be a challenge to go through the form and complete all the risk assessment elements”  “during the rush hours when there are more than one admission at a time. So going through the formalities of filling the risk assessment will be a little bit tedious” ( P15 R)  “sometimes admitting doctors are very busy and they are not able to do the risk assessment ( P6 SS) | 15 |
|  | If patient not admitted under my speciality, the risk assessment might be missed | “If it is my patient, I would. If the patients are not under me, I will not be doing the risk assessment. We can recommend.” ( P8 SS)  “If the patient was admitted under another specialty, surgical, we go through the assessment. We do not do it in the system, but we write our recommendation so implementing the scoring system, we leave it for the most responsible physician” (P9 S) | 9 |
|  | unavailability of mechanical prophylaxis could prevent ordering appropriate prophylaxis | “We have difficulty sometimes in applying mechanical DVT prophylaxis for patients on higher risk. I think there is a limitation in the number of the machines. So, we do order it, but it takes a quite some time” ( P9 S) | 3 |
|  | Availability of a VTE nurse/coordinator could facilitate the VTE risk assessment process | “the hospitalist can make sure that these guidelines are done and they can actually act as coordinators as well because they can call up different departments if the patient is managed by multiple departments” ( P8 SS)  “another professional or nurse could do the risk assessment and then if we just need to verify it then it would be easier for us” (P2 SS) | 7 |
|  | Electronic medical record will facilitate the implementation of VTE guidelines | “it is more simple because we have it on electronic medical record”( P8 SS)  “I think it is quite convenient now because with the electronic system everything is there. You only have to check select or deselect”( P3 S) | 13 |
| **Social influences** | I discuss the VTE recommendations with my team and other teams | “discussion with our Team”  “If there is something new to the patient then we will start to discuss and agree or disagree” ( P7 S)  “we take multidisciplinary decisions to make better care” (P13 S) | 12 |
|  | I might change the type of prophylaxis ordered based on my discussion with my seniors | “During the round for example while discussion with our consultants may be the type of the DVT prophylaxis will be changed” ( P7 S)  if my Senior tells me tells me I should do it and I should not mind it. Most of the time I agree because they are my seniors.  (P11 S) | 8 |
|  | If I need more information I refer to an expert in the field | “for difficult cases or challenging cases or ambiguous cases we usually refer back to the Hematologist” (P10 C)  “Sometimes if we are not really sure if we should give the prophylaxis or not, we might consult, other specialty like hematology” (P6 SS) | 7 |
|  | The family level of awareness and understanding of the VTE risks might affect patient from receiving the appropriate prophylaxis | “The family do not have this much of understanding of the risks though we explain to them” ( P5 SS)  “Sometimes there are patients who refuse, that affects your decision for ordering prophylaxis” ( P16 R)  “When the patient refuses treatment (prophylaxis) quite usually it is for specific reasons. We find that quite no one had explained to her why she needed to take that, but when we explain why she needs to take it. She was quite willing to go ahead. So again the refusal is usually related to patient education” ( P10 C) | 9 |
| **Behavioural regulation** | Monitoring the compliance to VTE guidelines and sharing the results will induce/ enforce the implementation of VTE guidelines. | “Leadership should monitor our compliance” ( P7 S)  “there should be a supervision” ( P5 SS) | 13 |
|  | Including the compliance to VTE guidelines in the physician’s performance evaluation process will induce/ enforce the implementation of VTE guidelines. | “if the administration wants to be very strict about it, maybe they have to include in the Individual performance evaluation” (P11 S) | 4 |
|  | Having VTE guidelines mandatory as a hospital policy will enforce the implementation of VTE guidelines. | “it is a part of the hospital policy which should be done” (P8 SS)  I mentioned mandating it in the system so the admission order would not be processed until the VTE assessment is done (P15 R) | 9 |
|  | Mandating the VTE guidelines as a hospital policy might affect the physicians role and autonomy | “when we have too much of the regulatory things to be done it does lead to physician burn out, when you say restrictive and make it mandatory physicians feel like you are taking away their autonomy”( P10 C) | 2 |
| **Nature of the behaviour** | I do VTE risk assessment for all patients | “Full intention. There is no hesitance” ( P1 C)  “for all my patients I do VTE risk assessment” ( P2 SS)  “any patient that gets admitted, we assess him for the VTE” ( P12 S) | 8 |
|  | I don’t do VTE risk assessment to all my patients | “It is not being 100% followed. (P6 SS)  “It is not that all the admitted patients is missed may be one or two patients” (P11 S) | 8 |
|  | I order sometimes prophylaxis without conducting VTE risk assessment | “most patients under the medical side are already started on prophylaxis even though the risk assessment has not been done” ( P11 S)  I am comfortable enough to start the DVT prophylaxis even without filling the scoring system (P14 SS) | 6 |
|  | I prescribe prophylaxis regardless of the risk score / I follow my clinical judgement | “If it is a young patient and unconscious, usually I am giving prophylaxis regardless of the score” ( P7 S)  “For most patients who are hospitalized, we start prophylaxis because we are not sure if he is going to stay long or not and then the MRP on the next day can reassess and decide. So we will take the safest approach. Even for unconscious patients we follow the same approach” ( P8 SS)  “ I follow my own judgment “( P8 SS) | 6 |
