# Supplementary material for: Physicians’ perceptions and preferences for implementing venous thromboembolism (VTE) clinical practice guidelines: a qualitative study using the Theoretical Domains Framework (TDF)
Source: Arch Public Health. 2022 Feb 15;80:52. doi: 10.1186/s13690-022-00820-7 (PMC8845331; doi:10.1186/s13690-022-00820-7)
Supplement: Supplementary file 5 — Additional file 5. Summary of belief statements and sample quotes assigned to the theoretical domains identified as not relevant. [file 13690_2022_820_MOESM5_ESM.docx]

| Summary of belief statements and sample quotes from physicians assigned to the theoretical domains identified as not relevant | | | |
| --- | --- | --- | --- |
| Key Domain | **Themes** | Sample Quote | Frequency |
| **Skills** | I am skilled enough to conduct the VTE risk assessment and order recommended prophylaxis | “it is part of patient’s general assessment” ( P5 SS)  “it takes good history taking skills, good physical examination skills and it should include a good clinical judgment and be able to decide” (P9 S)  “Basic medical background and detailed history taking and clinical assessment” ( P15 R) | 11 |
| **Social/professional role and identity** | It is my job to conduct VTE risk assessment and order the recommended prophylaxis | “It is my job”  “I want to make sure that my patient does not get any complication in the hospital. Discharging patients safe and sound” ( P9 S)  “It is part of our job” (P7 S) | 9 |
| **Optimism** |  |  | 0 |
| **Intentions** | I intend to prevent complications that might happen to patients | I am putting in my mind that I am preventing the patient from developing something serious in the hospital. This is most important ( P12)  I want to make sure that my patient does not get any complication in the hospital ( P9)  I know that I am giving my patients the best that they should have in whatever field (P10C) | 4 |
| **Memory, attention and decision processes** | VTE guidelines facilitates attention to detail steps to follow using a tool | “We have the VTE assessment form” ( P6 SS)  “It is a series of questions tick boxes that need to be done and then you provide the necessary prophylaxis” ( P10 C)  “We usually have an online chart for VTE risk in the admission package” ( P15 R) | 15 |
| **Emotion** | My emotions do not influence whether or not I conduct VTE risk assessment and order the recommended prophylaxis | “I do not feel nervous” (P14 SS)  “Emotions should not affect a physician’s decision” ( P10 C) | 8 |
|  | I am happy to implement the VTE guidelines | “I feel happy when I implement the guidelines because it has a lot for the patient’s safety in the hospital” (P1 C)  “For me as a physician, I feel happy and safe that I am preventing the patient from getting any life threatening condition or morbidity or mortality in the hospital” (P12 S) | 4 |
|  | I am satisfied to implement the VTE guidelines | “To satisfy my own conscience that you know that my patients are getting the best care and comply with the hospital guidelines” (P 10 C)  “I feel that I am satisfied about our own practice” (P9 S) | 6 |
