# Supplementary material for: Physicians’ perceptions and preferences for implementing venous thromboembolism (VTE) clinical practice guidelines: a qualitative study using the Theoretical Domains Framework (TDF)
Source: Arch Public Health. 2022 Feb 15;80:52. doi: 10.1186/s13690-022-00820-7 (PMC8845331; doi:10.1186/s13690-022-00820-7)
Supplement: Supplementary file 6 — Additional file 6. Barriers and facilitators. [file 13690_2022_820_MOESM6_ESM.docx]

| Key Domain | **Themes** | |
| --- | --- | --- |
|  | **Facilitators** | **Barriers** |
| **Knowledge** | Providing information about the importance of VTE guidelines | Unclear clinical practice guidelines recommendations for prophylaxis management in certain clinical conditions |
|  |  | Limited clinical information about the patient |
| **Beliefs about capabilities** | user-friendly VTE risk assessment tool including scores linked to order sets for prophylaxis |  |
|  | Training on the VTE risk assessment tool |  |
| **Beliefs about Consequences** | Provide information about the importance of VTE guidelines and health consequences | The risk of bleeding due to prophylaxis treatment in complicated cases |
| **Reinforcement** | Recognition from work context |  |
|  | Encouragement by senior physicians |  |
|  | Continuous reminders in the morning rounds and discussions during staff meetings |  |
| **Goals** | Adopt VTE prevention as a patient safety goal |  |
| **Environmental context and resources** | Automated VTE risk assessment tool integrated in the electronic medical record | Workload pressure and competing tasks |
|  | Availability of a VTE nurse/coordinator | Undefined responsibilities about completing the risk assessment |
|  |  | Unavailability of mechanical prophylaxis |
| **Social influences** | Multidisciplinary team approach | The family level of awareness and understanding of the VTE risks |
|  | Senior physicians role and support |  |
|  | An expert in the field |  |
| **Behavioural regulation** | Monitoring and providing evaluative feedback on performance to physicians |  |
|  | Setting outcome goals and including them in the performance evaluation |  |
|  | Having VTE guidelines mandatory as a hospital policy |  |
